# Escalating Doses of Buprenorphine for the Treatment of Buprenorphine-Induced Opioid Withdrawal

**DOI:** 10.7759/cureus.80527

**Published:** 2025-03-13

**Authors:** Steven J Laxton

**Affiliations:** 1 Department of Emergency Medicine, University of Tennessee Health Science Center (UTHSC) Nashville, Saint Thomas Health, Murfreesboro, USA

**Keywords:** buprenorphine, opiate withdrawal, opioid withdrawals, pain management, suboxone

## Abstract

This report outlines a case of opiate withdrawal that occurred following the initiation of treatment for opioid use disorder (OUD) with buprenorphine. Buprenorphine is a novel drug that is used in the treatment of OUD and is incredibly effective in doing so. However, a rare and paradoxical effect can occur when treatment is initiated with buprenorphine, where the drug, due to its unusual mechanism of action, precipitates withdrawal. Most of the time, withdrawal can be treated with supportive therapy. However, there is growing evidence in the literature that withdrawals precipitated by buprenorphine can and should be treated with increased doses of buprenorphine. This case is an example of this exact phenomenon. In this case, our patient had a longstanding OUD, particularly fentanyl use, which was treated the day of presentation with the initiation of buprenorphine. She later presented to the emergency department in opioid withdrawal, which was treated with adjunct therapy that did not aid in the resolution of symptoms. Finally, escalating doses of buprenorphine were administered, which resolved the patient’s symptoms, highlighting another case to be added to the growing evidence that opioid withdrawals precipitated by buprenorphine can and should be treated with escalating doses of buprenorphine.

## Introduction

Opioid use disorder (OUD) is a physical dependence that is created through the routine use of opioids, whether prescription or not. After developing physical dependence, opioid withdrawals can occur. There are many medications to treat OUD, including buprenorphine, which is highlighted in this report as an agent that can both precipitate and treat acute opioid withdrawals [1]. In the United States, the mainstay of treatment for OUD is either buprenorphine or methadone. Both are effective at treating OUD, but buprenorphine has a unique adverse effect of precipitating withdrawals.

OUD is rising in prevalence due to the increased potency of synthetic opioids (fentanyl being approximately 50-100 times more potent than morphine). Deaths due to opioid overdose are also increasing [2]. Along with the increase in potency of drugs, the number of synthetic opioid deaths has also increased by 426% and 79%, respectively, during the years 2013 and 2014 [3].

There exist many drugs used to treat OUD, and, similar to all drugs, there are side effects [4]. In this case report, a rare side effect of buprenorphine, a drug used in treating OUD, is presented. This side effect is subsequently treated using novel therapy, providing the same medication as the offending agent but in escalating doses until symptoms are resolved. This strategy has been previously described but not broadly implemented, whether due to clinician unfamiliarity with buprenorphine, concern that further doses will only escalate the symptoms of withdrawal, or patient hesitancy.

Regardless, this case adds an example of opioid withdrawals precipitated by buprenorphine treated with high-dose buprenorphine to aid clinicians in providing effective care for precipitated opioid withdrawal.

## Case presentation

The patient in this case is a 27-year-old female with a history of prior cocaine use, current OUD, and prior withdrawals from opioids. The patient presented to the emergency department with a chief complaint of “fentanyl withdrawals.” The history of the present illness was reported as a six-hour history of feeling “like I am withdrawing.” She experienced nausea and vomiting that was non-bloody and nonbilious, as well as diffuse abdominal discomfort and diarrhea that was non-bloody as well. She stated that she also felt sweaty and anxious. She stated that she had an extensive history of intermittent opioid withdrawals and that this felt very similar to prior episodes. She did report, however, that she had last used fentanyl approximately sixteen hours prior to arrival, and on the day of presentation, she had just started treatment for OUD. She was started on buprenorphine/naloxone at a dose of 8 mg/2 mg. She had taken a dose approximately 30 minutes prior to the onset of symptoms.

The patient had vital signs reported as heart rate 120, blood pressure 116/89, respiratory rate 28, oxygen saturation 98%, and weight 87 kg. Her physical exam was noted for anxiety, flushing of skin, tachycardia with normal rhythm, increased respiratory rate and clear, normal lung sounds, mild abdominal tenderness throughout without distension or rebound, and the patient was actively producing emesis. Laboratory data, including a complete blood count, comprehensive metabolic panel, and lipase, were normal, and a serum pregnancy test was negative.

The patient was initially treated with supportive medications, including 4 mg ondansetron IV repeated twice, 1.25 mg droperidol repeated twice, and 1000 mL lactated ringers intravenously. These were unable to resolve her symptoms. After a brief literature review and acknowledgment of a case similar to this in which withdrawals were treated with escalating doses of buprenorphine with success, the decision was then made to pursue this course of action. Two additional doses of buprenorphine/naloxone 8 mg/2 mg were administered for a total of 24 mg/6 mg total 24-hour dose. After the third dose, the patient had complete resolution of symptoms and could be discharged home. The patient had a follow-up scheduled with her addiction specialist the next day, which was reassuring as the patient could possibly require a continued elevated daily dose prior to tapering.

## Discussion

Opioid use disorder

OUD is a disorder characterized by a dependence on natural and synthetic substances that act at one of the main opioid receptors (mu, kappa, delta, nociceptin). The effect achieved by these substances can cause pain relief and sedation. A sense of euphoria can also be experienced through the ingestion of opioids.

Many opioids exist, all of which have the potential for addiction and misuse. To list a few, opium, from which morphine, heroin, and codeine are derived, and synthetic opioids, including oxycodone, hydrocodone, fentanyl, tramadol, and methadone.

The prevalence of opioid use has also increased. In the United States, historically, heroin was the mainstay drug abused in OUD; however, over the past decade, this has switched to fentanyl due to efficiency in production as well as increased potency, leading to increased addictive properties. The prevalence of OUD has increased nearly 4.5-fold in a recent study that captured data from 2010 to 2019 [4].

Patients who suffer from OUD certainly can have a spectrum of illness severity. That spectrum can range from maintaining a job and relationships to acute intoxication causing the patient to appear “drunk” or even as severe as overdosing, causing death from apnea and hypoxic respiratory failure. These patients can also present withdrawal symptoms, which will be discussed later in this article. Thus, a high index of suspicion should be held.

There exist many consequences downstream from OUD. Aside from the financial impact, there are also health consequences. Infection is one consequence and ranges depending on the route of ingestion. The highest risk for this remains among the patients that participate in IV use of opioids, where they can subsequently have bacterial seeding through improper cleaning of injection sites, abscess formation, endocarditis, pulmonary abscesses, etc. Other health effects that can occur include changes in bowel habits, which can range from constipation, most commonly, to diarrhea during withdrawals, as well as changes to pain reception, including an increased sense of pain when it occurs. The most severe health consequences remain, however, when a patient overdoses on opioids. This can be as severe as death due to apnea and hypoxia.

Current treatment of opioid use disorder

There are many treatments currently available for OUD. The treatments we will discuss here will only be pharmacological treatments as the case presented here is secondary to a pharmacological treatment for OUD. There exist many drugs for the treatment of OUD that work as an opioid antagonist (i.e., naltrexone) or agonist (i.e., buprenorphine).

Either therapy is effective [5]. However, there is definitive evidence that does show treatment via opioid agonist decreases mortality, thus favoring drugs such as buprenorphine or methadone [6]. Among the opioid agonists, buprenorphine is favored as it also contains additional advantages. A major advantage is that buprenorphine has a lower risk of death if a patient overdoses on it [6]. This is due to the mechanism of action of buprenorphine being a partial opioid agonist and causing decreased respiratory depression.

While both drugs perform well in the treatment of OUD, both have side effects. One that will be discussed in further detail is the unique side effect of precipitated opioid withdrawal that can occur with the administration of buprenorphine. Buprenorphine is a partial mu-opioid agonist that causes the displacement of full agonists at the mu-opioid receptor. The sudden shift from full to partial opioid agonism leads to withdrawal symptoms and/or uncontrolled pain. This side effect is uncommon and has yet to be quantified for its rate of occurrence. There are known risk factors for this condition. These include a high level of physical dependence, recent fentanyl or methadone use, recent benzodiazepine use, no prior buprenorphine use, a low initial dose of buprenorphine, chronic kidney disease, and cirrhosis. In the case presented in this article, the patient had four of these risk factors, placing her at high risk for precipitated opioid withdrawal.

Opioid withdrawals

Opioid withdrawals affect multiple organ systems, including the central nervous system, cardiovascular, respiratory, dermatologic, and gastrointestinal [7]. The most common symptoms and findings can include anxiety, tremors, dilated pupils, sweating, increased respiratory rate, tachycardia, hypertension, nausea, vomiting, diffuse abdominal discomfort, and diarrhea. These symptoms can be treated with non-opioid medications (i.e., anti-emetics, antipsychotics, benzodiazepines) for supportive therapy or with opioid agonists for management, both of which are acceptable practices with the end goal of suppressing the symptoms of withdrawal [8].

As this is a rare side effect of buprenorphine, it is difficult to estimate. A prior study estimated that up to 3% of the initiation of treatment for OUD with buprenorphine can experience this side effect [9]. Data does suggest, though, that buprenorphine is very effective in the treatment of OUD and withdrawal when compared to methadone and clonidine, thus favoring its treatment for use in the emergency department [10].

Another aspect that may increase the incidence of precipitated opioid withdrawal by buprenorphine is that it has been found that use of fentanyl is associated with a higher incidence of precipitated withdrawal than OUD secondary to other opioids [11]. As this phenomenon is not well characterized, it also lacks a well-defined description of the dose of buprenorphine that most effectively treats symptoms. Most reports have symptom resolution at 20 mg or 24 mg buprenorphine, as in this report [12]. However, some cases have reported much higher doses implemented for the management of precipitated opioid withdrawal using 63 mg in 24 hours [13,14]; however, this patient was managed in an inpatient hospital setting, which would be most appropriate if such high doses were needed. This case benefits from challenging the upper dose limit of buprenorphine, which has also been challenged previously.

## Conclusions

Buprenorphine is an effective drug used in treating OUD. This drug, however, can precipitate opioid withdrawals due to its unusual mechanism of action of partial opioid agonism. The case described in this article describes this rare side effect of buprenorphine and the ability to overcome the same side effect by escalating the dose and administering additional doses of buprenorphine.

Usually, opioid withdrawals are self-limiting and can be treated with supportive therapy and symptomatic management. In the case that this is unsuccessful, similar to the patient in this case report, then buprenorphine is another tool that physicians and clinicians can employ to aid in the management of opioid withdrawals.
